# Impact of the number of players on the emergence of creative movements in small-sided soccer games: a systematic review emphasizing deliberate practice

**DOI:** 10.3389/fpsyg.2023.1253654

**Published:** 2023-10-16

**Authors:** Lucas Shoiti Carvalho Ueda, Michel Milistetd, Gibson Moreira Praça, Gabriel Silveira Guedes da Maia, Juliano Fernandes da Silva, Paulo Henrique Borges

**Affiliations:** ^1^Department of Physical Education, Center of Sports, Federal University of Santa Catarina, Florianópolis, Brazil; ^2^Department of Sports, Federal University of Minas Gerais, Belo Horizonte, Brazil

**Keywords:** divergent thinking, creativity, game format, tactical creativity, exploratory behavior

## Abstract

**Introduction:**

Creativity is a recognized quality in various areas, including sports. Within the training processes, various modifications to objectives, game configurations, rules, among other factors, can be considered to favor creative solutions to the tactical problems inherent to soccer. This systematic review aimed to identify the impact of the number of players on the emergence of creative movements in small-sided soccer games, emphasizing deliberate practice.

**Methods:**

A systematic review of Scopus, PubMed, Scielo, PsycInfo, SportDiscus and Lilacs databases was performed according to PRISMA guidelines. Eligibility criteria were defined based on the elements of population, context and concept. Only full articles published in scientific journals written in English were included. No period restriction was applied.

**Results:**

Five papers were included and the results of studies indicate greater number of actions, variability, and creativity in small-sided games compared to formal soccer matches. When comparing different small-sided game formats, 5 v 5 showed higher values in terms of total number of actions compared to 7 v 7, and the absolute number of original and creative actions tended to decrease as the game format increased. Imbalanced small-sided games format can promote increased exploratory behavior. Structural manipulation in goal positioning in 5 v 5 games may also influence the originality of tactical behaviors, while the use of different ball types in 4 v 4 games appears to decrease fluency values. In 6 v 6 games, fluency and versatility are negatively impacted.

**Conclusion:**

Reduced game formats with fewer players and in smaller field dimensions provide more suggestive environment for exploratory behavior, variability and original and creative actions. The protocol was registered on the Open Science Framework (OSF) on 2 December 2022 (DOI: 10.17605/OSF.IO/VN6YZ).

**Systematic review registration:**

[https://osf.io/jmf4k/].

## 1. Introduction

Creativity is a quality required in various areas of expertise. Nevertheless, studies on creativity received greater attention only after Joy Paul Guilford’s historic speech upon assuming the presidency of the American Psychological Association (APA) in 1950 (De Sa Fardilha and Allen, 2019). In this speech and in his manuscripts, the president of APA pointed out the negligence of studies in the psychology field regarding the subject of creativity, as well as the importance of studying it rigorously and systematically (Guilford, 1950). From then on, creativity began to be understood as the manifestation of new and original ideas that are at the same time useful and designed for problem solving (Guilford, 1956; Sternberg and Lubart, 1999).

The “Guilfordian” view of the phenomenon proposes four theoretical constructs for recognizing creativity, namely fluency, flexibility, originality, and elaboration. Fluency refers to the generation of large number of ideas and responses in a short period of time; flexibility concerns the ability to think in different categories or perspectives, switching from one class of ideas to another; originality consists of the unique or rare quality of ideas; while elaboration involves expanding and coherently detailing the generated ideas (Furley and Memmert, 2018; Büning et al., 2020).

Advances in studies based on cognitive approaches to creativity have contributed to a paradigm shift and consequent shift away from innatist theories that have long supported discussions in this area (Ritter and Mostert, 2017). From then on, new theoretical perspectives have been considered. Amabile (1983) highlighted the importance of understanding social and motivational aspects beyond cognitive ones. In line with this, Glăveanu (2010) also attributes creativity to cultural and social factors, taking into account the available affordances in the context of action, i.e., social and cultural possibilities for an action to occur. Sharing a view that transcends the individual and isolated analysis of creative action, Csikszentmihalyi (1999) changes the origin focus of creation, shifting the genesis of the creative process from the psychological sphere and arguing that personal factors such as personality, intrinsic motivation, and values, while important, are not sufficient to explain creativity. By considering creativity as a process that emerges from interactions between the person, the symbol system, and the social organization of domain, Csikszentmihalyi presents a systemic model of creativity, in which the creative process is characterized by symbolic changes operationalized by individuals, embedded in a social domain, and with the participation of the field of expertise that validates and introduces innovations into society.

With the advancement of theoretical models capable of explaining the creative phenomenon, researchers in sports pedagogy have found scientific support to help them understand how the teaching and training processes of sports can contribute for the production of surprising, original, and flexible tactical-technical responses for a given game situation (Roth, 2005; Memmert and Roth, 2007). The Tactical Creativity Approach (TCA) model presents the 6 D’s to foster tactical creativity, which are: Deliberate Play, 1-Dimension Games, Diversification, Deliberate Coaching, Deliberate Motivation, and Deliberate Practice (Memmert, 2015). Although still incipient, some findings in literature suggest that deliberate play, free, informal, and unstructured game situations seem to favor the development of creativity in young players (Memmert et al., 2010; Roca and Ford, 2021).

In addition to the cognitive perspective of understanding the phenomenon, researchers have brought the understanding of creativity closer to the ecological approach, which consider the importance of the set of tasks, personal and environmental memories and also their interaction on the action system. In this sense, greater contextual variability and freedom of exploration increase the emergence of new, adaptive and functional solutions (Hristovski et al., 2011; Orth et al., 2017).

In soccer, the development of tactical-technical content and creativity through games can occur by manipulating the structural and/or functional rules of small-sided games (Santos et al., 2016; Duncan et al., 2022). In this sense, a study conducted by Santos et al. (2018) found that a training program with small-sided games, based on a wide range of modifications to objectives, game configurations, rules, and other factors has the potential to develop creativity in soccer, corroborating the findings of Coutinho et al. (2018), who found improvements after a training program based on differential learning for physical performance, technical variables and creative components. As previously seen, variability is an important indicator for exploration and functional solutions, Caso and van der Kamp (2020) compared different of small-sided game formats and concluded that the fewer players, the more (creative) actions they perform, finding that action variability in settings with fewer players may favor action originality.

Clemente and Sarmento (2020) demonstrated that small-sided games can be used to develop technical actions and skills in soccer. Another study showed that variations in games lead to different tactical behaviors (Clemente et al., 2021). However, no systematic review has sought to understand how the possible manipulations in small-sided games lead to the emergence of creative actions in soccer. Considering the importance of summarizing scientific evidence about small-sided game formats that favor creative actions in the teaching and training environment of soccer, the purpose of this systematic review was to understand the impact of the number of players and field dimensions on the emergence of creative movements in small-sided soccer games. The initial hypothesis of this investigation is that smaller game formats, with fewer participants, reduce the possibilities of interactions between teammates for problem-solving, increasing exploratory behaviors in the playing space that enable the emergence of new, original, useful and problem-oriented actions. The identification of which contexts favor the emergence of creative actions can provide coaches with insights to design training formats that generate environments with greater potential for creativity development. This quality, highly valued in the performance of soccer players, is found in admired and idolized athletes, such as Lionel Messi, whose skills have the potential to foster and influence the emergence of new creative players (Furley and Memmert, 2018).

## 2. Methods

### 2.1. Protocol and registration

The protocol and development of the systematic review followed the Preferred Reporting Items for Systematic Review and Meta-Analysis Protocols (PRISMA-P) 2015 (Moher et al., 2015), composed of a 17-item checklist intended to facilitate the preparation and reporting of the systematic review. The protocol was registered on the Open Science Framework (OSF) on 2 December 2022 (DOI: 10.17605/OSF.IO/VN6YZ).

### 2.2. Eligibility criteria

The eligibility criteria were defined based on the PCC mnemonic Population, Context, and Concept suggested by the Joanna Briggs Institute (Peters et al., 2015).

(I) Population: soccer players, without restrictions on age, sex, nationality, competitive level and practice time, aiming for greater coverage of the emerging theme.
(II) Context: small-sided games in soccer, covering all protocols that include the use of small-sided games, even if they involve other manipulations of task constraints in addition to the numerical configurations of the matches.
(III) Concept: assessment of creativity in soccer, bringing measures that can quantify the manifestation of creative movements in a clear and objective way.

At the time of the search, there was no restriction on the interval in years. Only full articles published in scientific journals written in English were included. For exclusion, some criteria were adopted based on PICOS (Methley et al., 2014).

(I) Population: not applicable. No criteria of exclusion were established for the population so as not to conflict with the eligibility criteria.
(II) Intervention: studies that evaluate creativity in small-sided games in other sports or those that are not related to small-sided soccer games, removing studies that involve small-sided games in sports other than soccer, as well as studies in soccer that use evaluation methods other than small-sided games.
(III) Comparison/Control: not applicable.
(IV) Outcome measure(s): studies in which the outcome is not related to the sports context or studies that did not present creativity measure, excluding studies that mentioned creativity without relating it to measures that allow clear and objective quantification.
(V) Types of studies: abstracts, thesis and dissertations, and qualitative studies.

### 2.3. Information sources and search strategy

The search was conducted from December 3 to 7, 2022, in electronic databases (Scopus, PubMed, Scielo, PsycInfo, SportDiscus, and Lilacs). This group of databases was chosen due to their relationship with the study theme and because the group includes bases used worldwide in review studies. The gray literature was not accessed in order not to contradict the exclusion criteria related with type of studies, and the additional search was performed through hand searches of reference list from included studies and email contact with experts. The keywords and Boolean operators for the search were: ‘creativity’ OR ‘intelligence’ AND ‘small-sided games’ OR ‘conditioned games’ OR ‘deliberate play’ OR ‘deliberate practice’ AND ‘soccer’.

### 2.4. Data management

The search results were exported from database websites such as ‘RIS’ files, a data exchange format used by a variety of reference managers, and inserted into the Covidence software for managing and streamlining systematic reviews.

### 2.5. Selection and data collection process

The systematic review selection process was presented in accordance with the PRISMA 2020 Flow Diagram for New Systematic Reviews proposed by Page et al. (2021). The process started with the identification, in which studies were found through the search strategy in databases and registers. Reports of selected data were generated using the Covidence reference manager software, the tool being applied so that reviewers could extract data independently and facilitating data export. Also, the tool was used to remove duplicate records before the screening phase. The study selection process was carried out by two independent reviewers. Reviewers 1 and 2 performed a calibration exercise before starting the independent analysis of screened records, whose task consisted of independently reading the titles and abstracts of 10 articles and subsequent discussion.

The first step of the screening phase consisted of reading the titles and abstracts of all articles found based on established search strategy in selected databases and that passed the duplicate filter. Subsequently, the two reviewers evaluated the articles with the intention of including them for the next stage or discarding them, respecting the eligibility criteria. After this process, the reviewers analyzed the disagreements and try to reach consensus. In cases where consensus was not possible, reviewer 3 was available to assist in the decision and made the final decision. Once again, a calibration was performed between reviewers 1 and 2, this time based on the reading, checking and discussion of three complete articles. The second step of the screening phase was operationally similar to the previous step. However, this time reviewers will read the articles that reached this stage in full and again differences were solved by reviewer 3.

Subsequently, the additional search step was performed in two ways. The first in the list of bibliographic references used in the included studies, to find possible studies that were not identified in the initial search. The second way, through email contact with the main authors in the area.

Finally, the reviewers met once again to seek a consensus and definition of selected articles. In this final stage of the selection process, only studies included in the review remained.

### 2.6. Data items

For the current study, fluency, originality and flexibility measures were selected as the main outcomes. These variables were chosen because they are often used to operationalize tactical creativity, identified by mean of factor analysis (Guilford, 1967). In this sense, originality is understood from the exceptionality of tactical solutions and can be rated by experts, flexibility encompasses the variety of tactical solutions, being determined by the diversity of actions/responses of test participants, and fluency concerns the number of tactical solutions that the individual generates for a specific situation of the match (Memmert, 2015). Exploratory behavior was also defined as an outcome measure, being understood as the “subsequent performance of a large number of movement configurations that reveal the hierarchical action landscape under specific constraints of each performer” (Hristovski et al., 2011, p. 187) or team.

### 2.7. Outcomes and prioritization

All included studies used more than one small-sided game format; however, only one analyzed the isolated impact of the balanced number of players and pitch size on creativity. Four other studies presented small-sided game structures, but also performed other manipulations. Two studies proposed imbalanced games, in which the number of players from the teams were different (Torrents et al., 2016; Canton et al., 2019). One of studies added the change in the positioning of goals (Canton et al., 2020) while another used different types of ball in games (Santos et al., 2020). Thus, in addition to the main outcomes related to the impact of pitch size and number of players on creativity measures, results related to other types of structural manipulations were also identified.

### 2.8. Risk of bias in individual studies

The Joanna Briggs Institute Critical Appraisal tools (JBI) for use in systematic reviews of cross-sectional studies (Moola et al., 2020) was used (Table 1). As recommended by PRISMA (Moher et al., 2015), two reviewers independently assessed each study based on the criteria used to rank risk of bias. Disagreements were resolved by consensus between reviewers 1 and 2, and consultation with a third reviewer was not necessary. The instrument consists of the following questions: “Were the inclusion criteria in the sample clearly defined?”; “Were the study subjects and the setting described in detail?”; “Was the exposure measured in a valid and reliable way?”; “Were standard criteria used for condition measurement objective?”; “Were confounding factors identified?”; “Were strategies to deal with confounding factors stated?”; “Were the outcomes measured in a valid and reliable way?”; “Was appropriate statistical analysis used?.” To classify questions, they were flagged as “yes,” “no,” “unclear” or “not applicable.”

**Table 1:** Characteristics of studies.

Authors (year)	Country	Sample	Context	Game format	Rules	Procedure	Measure of creativity	Primary outcomes
Torrents et al. (2016)	ESP	22 professionals ♂ Mean age of 25.6 years (SD = 4.9); 22 amateurs ♂ Mean age of 23.1 years (SD = 0.7).	Professional males from a single soccer and amateur male players enrolled in a sports sciences degree.	4 v 3 4 v 4 4 v 7 (40 × 30 m)	All SSG: official rules. In order to avoid the effect of the scoring, the scoreboard turned to 0 when any team achieved two goals.	2 × 3 min for each game format.	Notational analysis. Observation instrument adapted from Owen et al. (2014) and Costa et al. (2011).	Players seem to show more exploratory behavior when playing with numerical disadvantage.
Canton et al. (2019)	ESP	15 ♂ Under the age of 23 years. Mean age of 19.9 years (SD = 1.6); 15 ♂ Under the age of 15 years. Mean age of 13.8 years (SD = 0.4).	Each age group played in the same team and category. Under 23 group: Spanish 3rd division; Under 15 group: División de Honor, top level of the Spanish football league system of that age.	Balanced SSG: 4 v 4 (40 × 45 m); Imbalanced SSG: 4 v 4 5 v 4 4 v 5 6 v 4 4 v 6 (40 × 45 m)	Balanced SSG: fixed number of opponents. Imbalanced SSG: numerical change as follows: minute one: 4 vs. 4; minute two: 5 vs. 4; minute three: 4 vs. 5; minute four: 6 vs. 4; and minute five: 4 vs. 6. All SSG: official rules, except off-side and throw-ins. Goal kick reposition after a goal and throw-in.	2 × 5 min for each game format.	Positional data analysis.	The manipulation of the number of teammates and opponents at 1 min intervals promoted, in the Under 15, a slight increase in the exploratory behavior in both short- and long-term exploration breadth. In the Under 23, the same constraint promoted an unclear increase in the short-term exploration, and a very large increase in the long-term.
Canton et al. (2020)	ESP	24 ♂ Under the age of 12 years. Mean age of 11.3 years (SD = 0.8).	High-level soccer school and all of them had more than 1 year of experience in this school.	5 v 5 (31 × 37 m)	Three different situations of 5 v 5 SSG (front goals; right diagonal goals; and left diagonal goals).	6 × 5min for each goal positioning.	Positional data analysis.	Changing the positioning of goals in SSG in soccer modifies the originality of tactical behavior but does not seem to increase fluency and flexibility.
Caso and van der Kamp (2020)	NLD	24 ♂ 17 to 32 years. Mean age of 21.3 years (SD = 3.46).	Professional players affiliated with the same elite European soccer club and playing for their national team.	5 v 5 and 6 v 6 (36 × 18 m); 7 v 7 (54 × 18 m); 11 v 11 (105 × 64 m).	All SSG: official rules, except throw-ins. Goal kick repositions in these cases. 11 v 11 official match rules.	1 × 10 min for each game format.	Notational analysis. A score sheet was developed listing the definitions of creative soccer actions.	Players produced more creative actions in the three SSG-formats than in the 11-aside match. The number of original and creative actions seem to reduce increasing the pitch size, with none appearing during the 11 v 11.
Santos et al. (2020)	PRT	12 13 and 14 years. Mean age of 13.7 years (SD = 0.5).	Youth players with 6.1 ± 0.9 years of experience in soccer practice. All players were members of the same team.	4 v 4 (50 × 35 m); 6 v 6 (64 × 43 m).	Different balls were used in each game format and period of game (soccer, handball, rugby and mixed).	4 × 6 min for each game format, being the first bout using soccer ball, the second with handball, the third with rugby ball, and the last bout changing the ball type for each 2 min.	Creativity Behavior Assessment in Team Sports (CBATS).	4 v 4: fluency decreased with rugby ball compared to the soccer ball. 6 v 6: fluency and versatility decreased with the handball and rugby ball compared to the soccer ball; and fluency decreased with mixed balls compared to the soccer ball.

### 2.9. Confidence in the cumulative evidence

The assessment of confidence in the cumulative evidence was also carried out by two independent reviewers in order of verifying the strength of the body of evidence so that any disagreements are resolved through a consensus meeting or with the help of an expert.

The modified version of the Quality Index (Downs and Black, 1998) adopted in recent systematic reviews (Bujalance-Moreno et al., 2019; Praça et al., 2022) was used to assess the methodological quality of eligible studies. The original scale is composed of 27 items, of which only 14 were verified in studies, since the other criteria were considered not applicable to studies of this review. The tool modification resulted in the following criteria: (1) Is the hypothesis/aim/objective of the study clearly described?; (2) Are the main outcomes to be measured clearly described in the Introduction or Methods section?; (3) Are the characteristics of participants included in the study clearly described?; (6) Are the main findings of the study clearly described?; (7) Does the study provide estimates of the random data variability for the main outcomes; (10) Have current probability values been reported (e.g., 0.035 rather than <0.05) for the main outcomes except where the probability value was less than 0.001?; (11) Were subjects asked to participate in the study representative of the entire population from which they were recruited?; (12) Were subjects who were prepared to participate representative of the entire population from which were they recruited?; (15) Was an attempt made to blind those measuring the main intervention outcomes?; (16) If any of the results of studies were based on “data dredging,” was this made clear?; (18) Were the statistical tests used to assess the main appropriate outcomes?; (20) Were the main outcome measures used accurate (valid and reliable)?; (22) Were study subjects in different intervention groups (trials and cohort studies) or were cases and controls (case–control studies) recruited over the same period? (23) Were study subjects randomized to intervention groups?

### 2.10. Data synthesis

Studies were quantitatively synthesized and characterized by criteria of authors, year of publication, country, sample (number of participants, gender, and age) context (competitive level), game format, rules and procedure of the game, measure of creativity used, and primary outcomes. An additional analysis proposal was established by the authors for the identification of the area (m2) per player, bringing even more detailed data on the formatting of game spaces and offering more data for the planning of training processes based on reduced games in soccer aimed at the emergence of creative actions.

## 3. Results

### 3.1. Study selection

**Figure 1:**
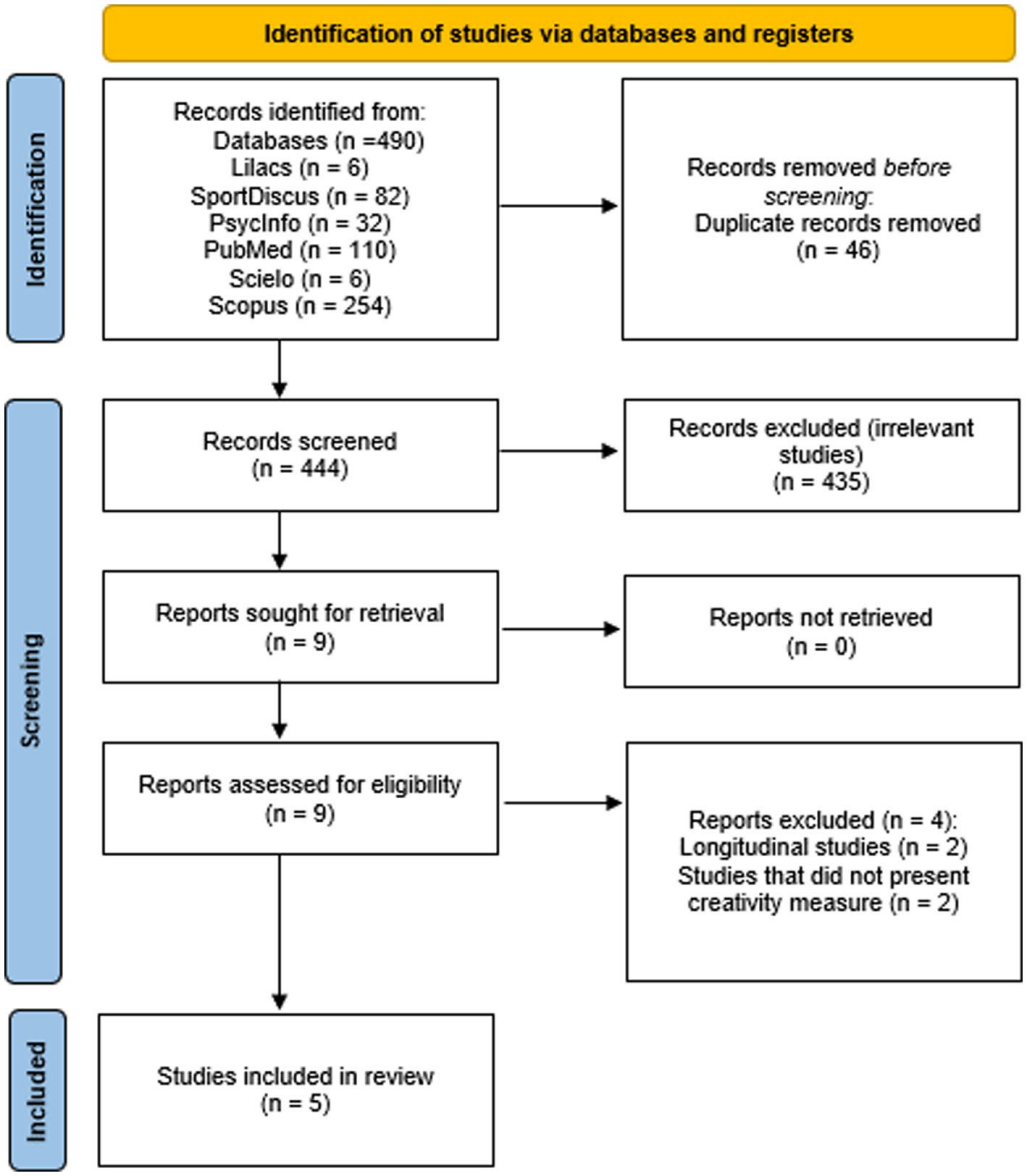
PRISMA flow diagram highlighting the selection process for the studies included in the current systematic review (Adapted from Page et al., 2021).

From main electronic databases searches, a total of 490 references were identified. After duplicated studies had been removed, 444 records remained. In the first screening step, 444 were assessed, and 9 were considered eligible for full-text reading. In the second step, 4 were excluded, two for not having cross-sectional design and two for not presenting creativity assessment measures in results. Therefore, only 5 papers met the inclusion criteria and were considered for qualitative synthesis. Within population, intervention, comparison, outcome and type of study specifications, no additional studies were found. The complete process of identification and selection of studies is provided in Figure 1.

### 3.2. Study characteristics

All included papers were classified as analytical cross-sectional studies according to the JBI reviewers manual, with the inclusion of a total of 134 participants. Studies were conducted in Spain (Torrents et al., 2016; Canton et al., 2019, 2020), Netherlands (Caso and van der Kamp, 2020) and Portugal (Santos et al., 2020) and were published between the years 2016 and 2020. In addition, only one study does not specify the gender of participants (Santos et al., 2020), while the other two are composed of male athletes (Torrents et al., 2016; Canton et al., 2019, 2020; Caso and van der Kamp, 2020). As for the context, two studies were composed of young soccer players (Canton et al., 2020; Santos et al., 2020); one was carried out with professional athletes (Caso and van der Kamp, 2020); another used two groups: amateur players enrolled in a sports sciences degree and professional athletes (Torrents et al., 2016); finally, one study used two groups of age categories: U-23 and U-15 (Canton et al., 2019). The practice time in the modality was explained in two of the three articles (Canton et al., 2020; Santos et al., 2020), being different between them.

With regard to procedures used to carry out games, the total time for each format and game specifications within studies varies between 6 and 30 min. One study analyzed the first 10 min continuously (Caso and van der Kamp, 2020), while the others divided them into different time periods (Torrents et al., 2016; Canton et al., 2019, 2020; Santos et al., 2020).

The instruments used to measure creativity ranged from analysis of positional data obtained through GPS equipment (Canton et al., 2019, 2020), notational analysis from a pre-established matrix for creative actions (Caso and van der Kamp, 2020), from a pre-prepared spreadsheet entitled Creativity Behavior Assessment in Team Sports (CBATS) (Santos et al., 2020) and from adapted observational instrument (Torrents et al., 2016). More information about study characteristics is provided in Table 1.

### 3.3. Risk of bias of studies

According to the JBI checklist for cross-sectional studies, all studies included at least 5 of the 8 items proposed in the instrument. Regarding item 1, three studies were not clear in describing the inclusion criteria (Canton et al., 2019, 2020; Santos et al., 2020). As for the standardization of the combination of groups, four studies made clear the use of a subjective division criterion based on the coach’s decision (Torrents et al., 2016; Canton et al., 2019, 2020; Santos et al., 2020), while one does not determine the final criterion for this division choice of team composition (Caso and van der Kamp, 2020). The criteria referring to confounding factors were considered not applicable to the five studies (items 4 and 5). The full assessment of the risk of bias in individual studies is provided in Table 2.

**Table 2:** Assessment of the risk of bias in individual studies.

Authors	Q1	Q2	Q3	Q4	Q5	Q6	Q7	Q8	Quality assessment
Torrents et al. (2016)	✓	✓	✓	✓	N/A	N/A	✓	✓	6
Canton et al. (2019)	U	✓	✓	✓	N/A	N/A	✓	✓	5
Canton et al. (2020)	U	✓	✓	✓	N/A	N/A	✓	✓	5
Caso and van der Kamp (2020)	✓	✓	✓	U	N/A	N/A	✓	✓	5
Santos et al. (2020)	U	✓	✓	✓	N/A	N/A	✓	✓	5

Q1. Were the inclusion criteria in the sample clearly defined? Q2. Were the study subjects and the setting described in detail? Q3. Was the exposure measured in a valid and reliable way? Q4. Were standard criteria used for measurement of the condition objective? Q5. Were confounding factors identified? Q6. Were strategies to deal with confounding factors stated? Q7. Were the outcomes measured in a valid and reliable way? Q8. Was statistical analysis used appropriate? ✓ - Yes; −- - No; U – Unclear; N/A – Not/Applicable. (Moola et al., 2020).

### 3.4. Results of individual studies

Torrents et al. (2016) sought to verify how restrictions arising from changes in the number of opponents and teammates affect the exploratory behavior of 22 professional players and 22 amateurs, in 4 v 3, 4 v 5 and 4 v 7 reduced games, on a field with dimensions of 40 × 30 m. The two amateur teams that played with fixed number of 4 players, called AMAa and AMAb, showed effects of the number of opponents. By analyzing the exploration values between different small game formats, AMAa showed small effect of the number of opponents when comparing games with 5 and 7 opponents. AMAb showed strong effects of the number of opponents when comparing 3 and 5 opponents, and also between 3 and 7 opponents. The two professional teams with fixed number of players, named PROa and PROb, showed moderate effects when comparing games with 3 and 5 opponents, and between 3 and 7 opponents. In the case of variable teams, playing with seven teammates clearly produced lower exploratory breadth compared with the other conditions. All teams showed strong effects of the number of teammates when comparing 5 and 7 teammates, and 3 and 7 teammates.

Canton et al. (2019) verified exploration rate and breadth values for temporary numerical balanced and unbalanced numerical conditions for each age group and SSG condition for two distinct age groups. For the age group under 23 years, increase in exploratory breadth was observed and the exploration rate showed unclear effects. For the under 15 age group, the mean exploratory breadth value clearly decreased using temporary numerical imbalances from an equilibrium situation. The exploitation rate reported that its average value would likely be reduced from a numerical equilibrium situation to a temporary numerical imbalance situation, with small effect size.

Canton et al. (2020) assessed 24 athletes (aged under 12 years) in three different situations of small-sided games with a 5 v 5 configuration, in which goals were positioned in front, diagonally to the right, and diagonally to the left relative to the direction of the attack of teams, and the field dimensions were set at 37 × 31 m. Based on the analysis of principal components extracted from the metrics observed through positional data analysis obtained by GPS tracking, it was observed that teams perform their behaviors differently depending on the type of structural constraints of the game. When positioning the goals diagonally, there is variation in the measures of some components, such as team length and width, centroid angle, distance from the centroid to the own goal, and sectors and corridors traveled. In this sense, tasks can be proposed to achieve training objectives related to spatial organization or tactical behaviors based on the practice of unusual scenarios, these atypicalities being related to an environment that favors originality.

Caso and van der Kamp (2020) examined variability and creativity in small-sided games of 5 v 5 (36 × 18 m), 6 v 6 (36 × 18 m), 7 v 7 (56 × 18 m), as well as formal 11 v 11 (105 × 64 m) games in a group of 24 professional players aged 17–32 years affiliated with the same elite European soccer team. Analysis of variance in the number of actions revealed significant effects of game format, and descriptive statistics show that the smaller the game format, the greater the total number of actions performed by players. Furthermore, *post-hoc* analyses indicated significant differences in favor of all small-sided game formats compared to the 11 v 11 game, and also in favor of 5 v 5 compared to 7 v 7. Regarding variability, the effect of game format was observed, with *post-hoc* analyses detecting the production of actions from more categories in all small-sided game formats compared to the formal game. Originality was recognized from categories of actions that were exclusively produced by one or two players (approximately 5% of participants), resulting in 14 actions from 6 distinct categories of actions. Of these actions, 10 were considered creative and appropriate. Descriptive statistics recognized higher total number of original and creative actions in smaller game configurations; however, inferential statistics could not be performed due to the low number of actions.

Santos et al. (2020) identified creative components incorporated into technical skills in 4 v 4 (50 × 35 m) and 6 v 6 (64 × 43 m) small-sided games with manipulation of ball type in a group of 12 young players from the same team. When compared, fluency using a rugby ball within both formats of small-sided games was considerably reduced compared to the use of a soccer ball. Additionally, in the 6 v 6 formats, fluency also decreased significantly when comparing the use of a soccer ball with that of a rugby ball, and there may have been a decrease in fluency in the comparison between a soccer ball and a handball. Finally, the versatility component also decreased when comparing the soccer ball to the handball and rugby balls.

### 3.5. Confidence in cumulative evidence

According to evaluation based on the adaptation of the critical appraisal instrument Quality Index (Downs and Black, 1998), studies by Torrents et al. (2016) and Canton et al. (2019) met 9 of the 14 items used (64.29%), while Canton et al. (2020) and Santos et al. (2020) met 8 (57.14%) of the items. Finally, the work by Caso and van der Kamp (2020) met 10 (71.43%) of the items. Further details regarding the evidence appraisal are available in Table 3.

**Table 3:** Critical appraisal of studies includes in systematic reviews.

Authors (year)		Criteria															
		1st	2nd	3rd	6th	7th	10th	11th	12th	15th	16th	18th	20th	22nd	23rd	*n*	%
Torrents et al. (2016)		1	1	1	1	1	0	0	0	0	1	1	1	1	0	9	64.29
Canton et al. (2019)		1	1	1	1	1	0	0	0	0	1	1	1	1	0	9	64.29
Canton et al. (2020)		1	1	0	1	1	1	0	0	0	1	1	1	0	0	8	57.14
Caso and van der Kamp (2020)		1	1	1	1	0	1	0	1	0	1	1	1	1	0	10	71.43
Santos et al. (2020)		1	1	0	1	1	0	0	0	0	1	1	1	1	0	8	57.14
Total	*n*	5	5	3	5	4	2	0	1	0	5	5	5	4	0		
	%	100.00	100.00	60.00	100.00	80.00	40.00	00.00	20.00	00.00	100.00	100.00	100.00	80.00	00.00		

1st: Is the hypothesis/aim/objective of the study clearly described? 2nd: Are the main outcomes to be measured clearly described in the Introduction or Methods section? 3rd: Are the characteristics of participants included in the study clearly described? 6th: Are the main findings of studies clearly described? 7th: Does the study provide estimates of the random data variability for the main outcomes? 10th: Have current probability values been reported (e.g., 0.035 rather than < 0.05) for the main outcomes except where the probability value was less than 0.001? 11th: Were subjects asked to participate in the study representative of the entire population from which they were recruited? 12th: Were subjects who were prepared to participate representative of the entire population from which were they recruited? 15th: Was an attempt made to blind those measuring the main intervention outcomes? 16th: If any of the results of the study were based on “data dredging,” was this made clear? 18th: Were the statistical tests used to assess the main appropriate outcomes? 20th: Were the main outcome measures used accurate (valid and reliable)? 22nd: Were study subjects in different intervention groups (trials and cohort studies) or were cases and controls (case–control studies) recruited over the same period? 23rd: Were study subjects randomized to intervention groups? 0 – No/Unable to determine; 1 – Yes (Downs and Black, 1998).

### 3.6. Additional analysis

In order to make the graphic representation of the additional analysis regarding the field area (m2) per player more accessible, the values of evaluated creativity components were converted to a scale from 0.00 to 10.00, assigning “10.00” to the highest value in each study and calculating the remaining values proportionally.

**Figure 2:**
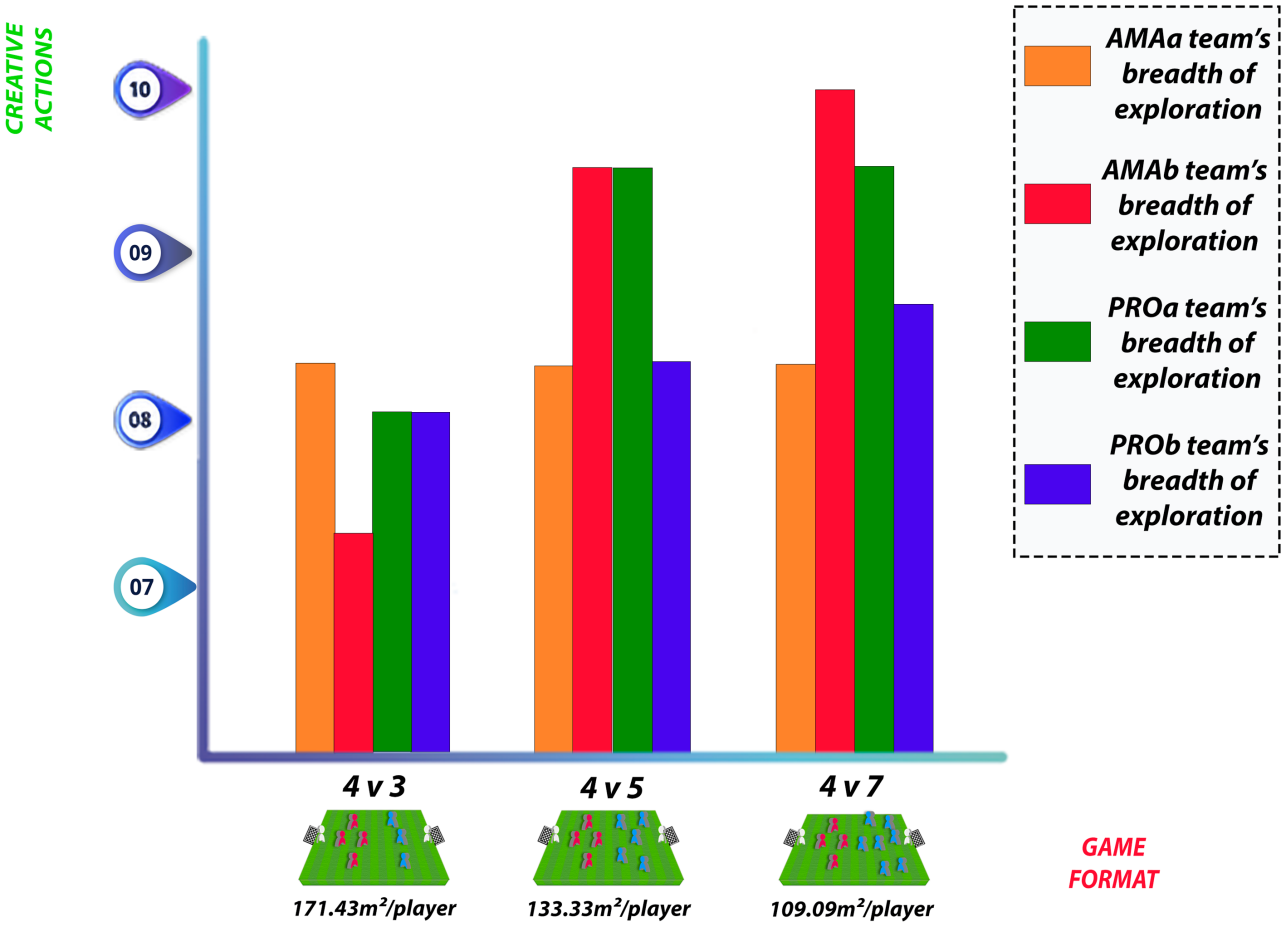
Breadth of exploration of teams for different relative playing areas.

Torrents et al. (2016) used unbalanced small-sided games with four fixed teams of 4 players (AMAa; AMAb; PROa; PROb) against teams with 3, 5, and 7 opponents (AMAc; AMAd; PROc; PROd). The 4 v 3, 4 v 5, and 4 v 7 game formats corresponded to relative areas of 171.43 m2/player, 133.33 m2/player, and 109.09 m2/player, respectively. The aforementioned authors relate the increase in exploratory behavior to the numerical relationship between teams, with the disadvantaged teams exhibiting higher exploratory behavior. Furthermore, the additional analysis of the present study demonstrates that in addition to the numerical disadvantage, the relative playing area can also be an indicator. Performing the proposed scalar conversion, the game format with 171.43 m2/player presented values of 8.42 for AMAa, 7.27 for AMAb, 8.00 for PROa and PROb. As for the game played in a space with relative area of 133.33 m2/player, AMAa (8.42), AMAb (9.41), PROa (9.41), PROb (8.42) start to show higher exploratory behavior values. Finally, by further reducing the relative playing area (109.09 m2/player), the trend of increasing the breadth of exploration remains for teams, with AMAa showing value of 8.42, AMAb 10.00, PROa 9.41, and PROb 8.88 (Figure 2).

**Figure 3:**
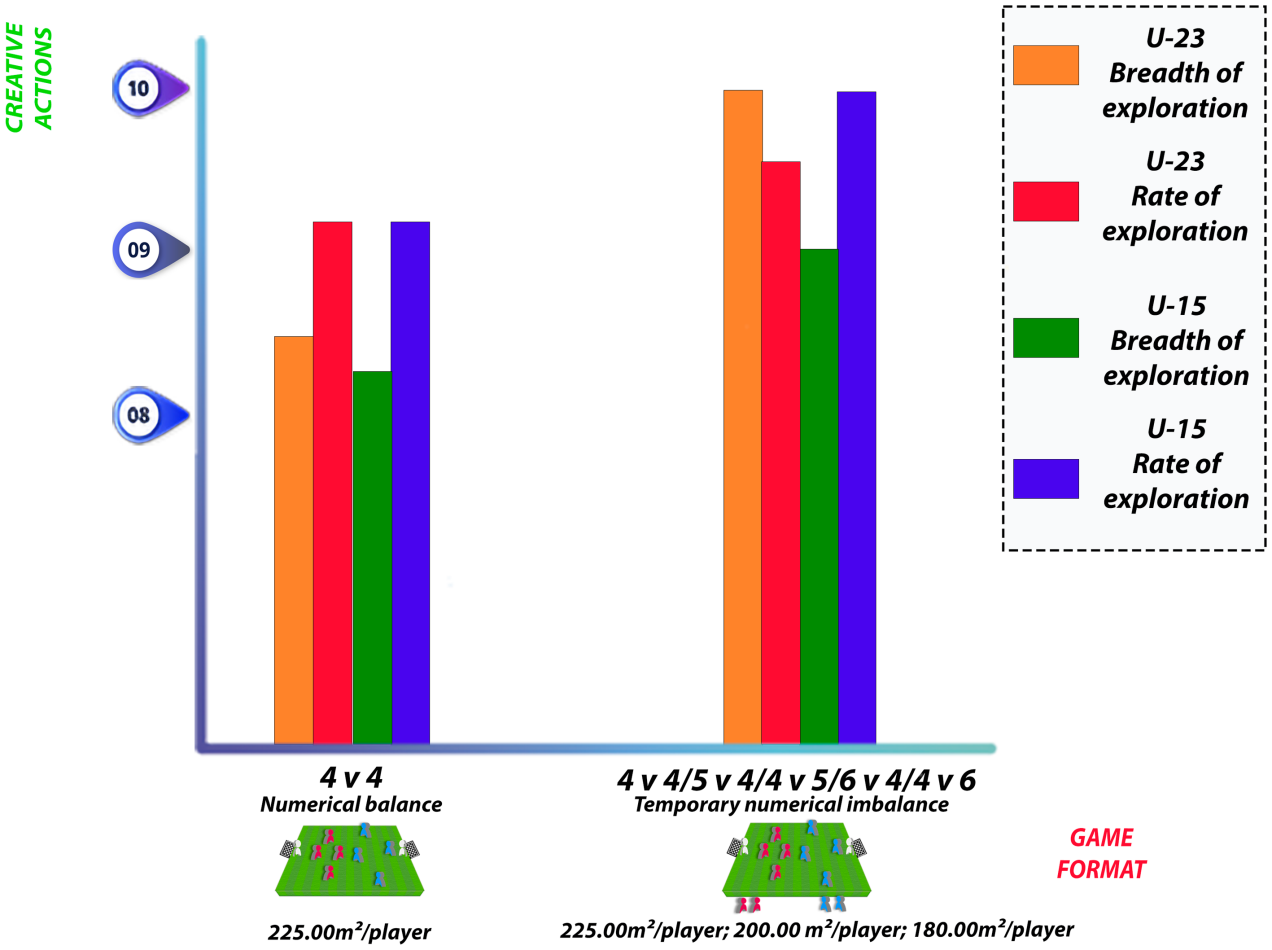
Rate and breadth of exploration for balanced and unbalanced game formats.

Canton et al. (2019), when analyzing balanced games (4 v 4) and unbalanced games where the number of players involved in the match changed every minute (min 1: 4 v 4; min 2: 5 v 4; min 3: 4 v 5; min 4: 6 v 4; and min 5: 4 v 6), also found increase in exploratory behavior for unbalanced matches. In this regard, it is worth noting that the alternation of players in small-sided games causes changes in the relative area per player over time, as it is reduced with each change in the game format (Figure 3).

**Figure 4:**
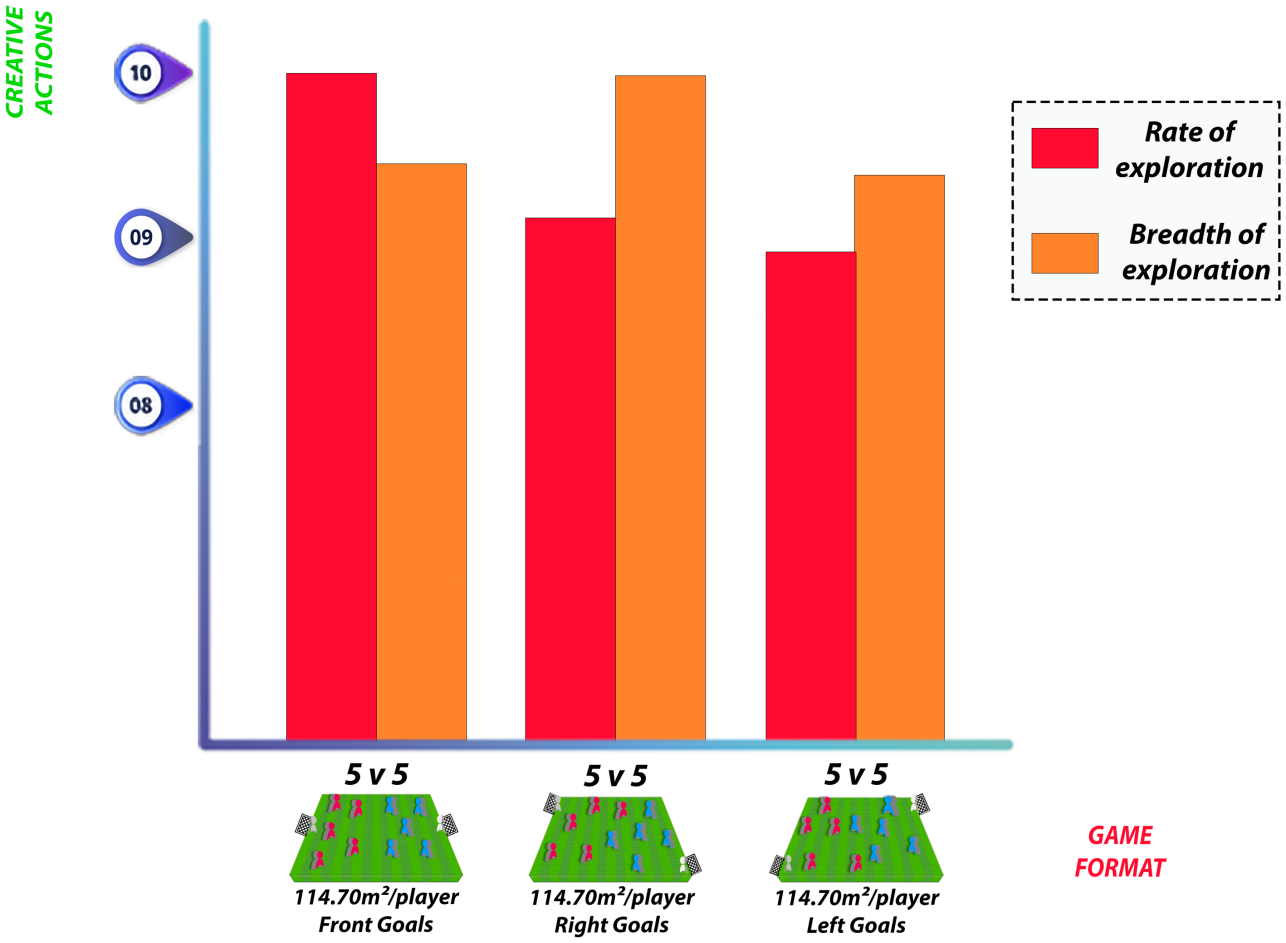
Exploration rate and exploration breadth for relative area of 114,70 m^²^/player.

Canton et al. (2020) used the same configuration regarding the field dimensions and number of players, manipulating only the positioning of goals. For a 5 v 5 game in which field measurements were 31 × 37 m, the relative area was 114.70 m2 per player. Regarding the proposed scale conversion, the game with front goals presented higher exploration rate values (10.00), followed by goals to the right (9.15) and goals to the left (8.94). For the exploration breadth, the results observed for goals to the right were higher (10.00) when compared to the front position (9.46) and to the left position (9.40) of goals (Figure 4).

**Figure 5:**
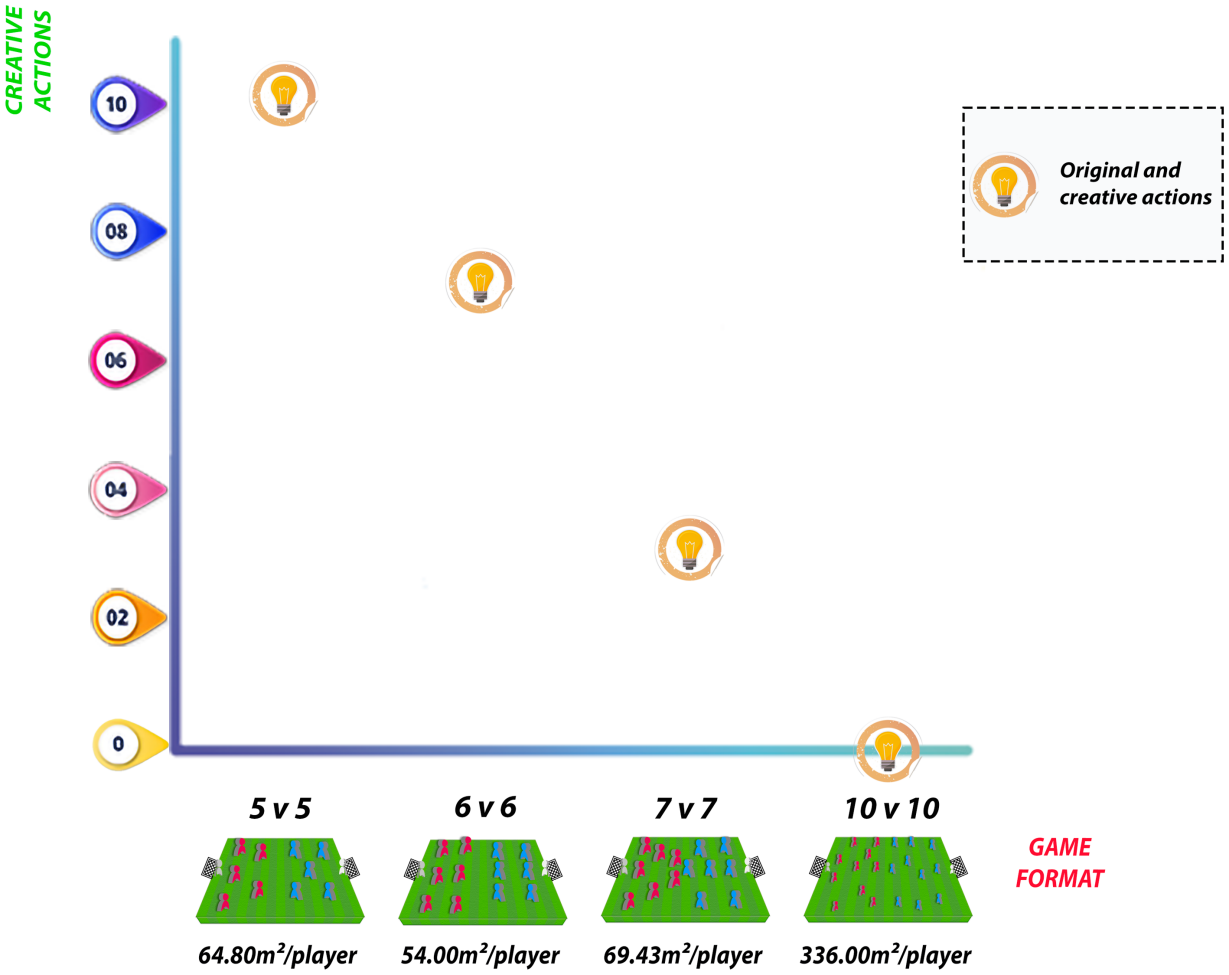
Original and creative actions for different relative areas per player.

Caso and van der Kamp (2020) used several game configurations, with different field dimensions and number of players. The 5 v 5 game played on a 36 × 18 m field represented a relative area of 64.80 m2/player and had the highest value of original and creative actions (10.00). When playing 6 v 6 on the same field (36 × 18 m), the relative area decreased to 54.00 m2/player and the proportional value assigned was the second highest (6.67). For larger game formats, the 7 v 7 on a 54 × 18 m field (relative area of 69.43 m2/player) showed a decrease in creative actions (3.33), while the 10 v 10 on a 105 × 64 m field (relative area of 336.00 m2/player) had no creative actions at all (0.00) (Figure 5).

**Figure 6:**
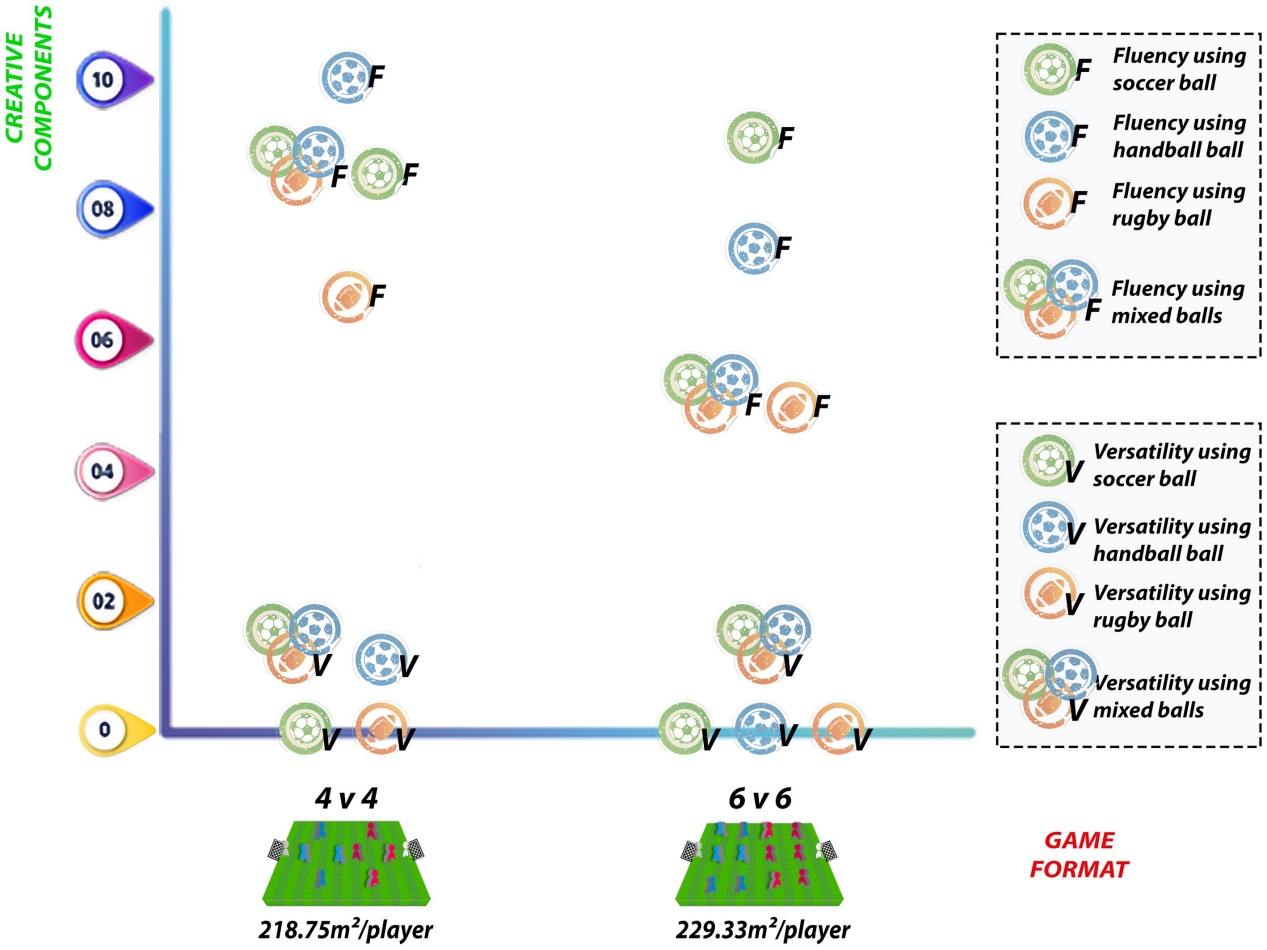
Fluency and versatility in different relative areas and with ball type manipulation.

The original study by Santos et al. (2020) did not propose a comparison between game formats, thus no inferential statistics were performed to analyze whether there are differences in creativity measures between 4 v 4 and 6 v 6 matches. However, considering the descriptive analysis, it is possible to observe that the median fluency and versatility values for each type of ball used either remained the same or decreased as the number of players and relative area per player increases, except for the game that used only the soccer ball. Assigning the proposed conversion in this additional analysis to the aforementioned study, the 4 v 4 game with dimensions of 50 × 35 m (relative area per player of 218.00 m2/player) obtained better values for the fluency component than the 6 v 6 game with dimensions of 64 × 43 m (relative area per player of 229.33 m2/player) using handball (10.00 vs. 7.50), rugby ball (6.67 vs. 5.00), and mixed ball (8.33 vs. 5.00), with the larger format only having an advantage with the soccer ball (8.33 vs. 9.17). On the other hand, versatility only presented different values between the 4 v 4 and 6 v 6 when using handball (1.67 vs. 0.00), also in favor of the smaller game format (Figure 6).

## 4. Discussion

This review aimed to investigate the impact of the number of players on the emergence of creative movements in small-sided soccer games, emphasizing studies carried out in a deliberate practice context. We hypothesized that smaller game formats, with fewer participants, reduce the possibilities of interactions between teammates for problem-solving, increasing exploratory behaviors in the playing space that enable the emergence of new, original, useful and problem-oriented actions. Following this reasoning, other manipulations that favor an increase in exploratory behavior could also contribute to the emergence of creativity. The present review found five articles that met the criteria, which is a low number with high heterogeneity in protocols, having very distinct creativity procedures and measures among them. This fact prevented the conduction of a meta-analysis and indicates the need for more research on the topic. The results found in the present study confirmed the expectation that the number of players impacts creativity measures, supporting the idea that smaller games favor the emergence of creative actions compared to formal matches. Additionally, it was also identified that structural changes in small-sided games can generate unusual environments in which different tactical responses may favor originality in actions. However, concerning the manipulation of different ball types, values related to creativity variables did not improve when using different balls.

### 4.1. Creativity measurement

The fact that the present review found studies that used different methods to measure creativity is also due to the epistemological possibilities of understanding the phenomenon, which can be translated through the choice of creativity instruments and measures to be evaluated. In the cognitive perspective, studies on creativity in sports follow the precepts of psychology, appropriating and presenting the fluency, flexibility, and originality constructs. Studies using this perspective for small-sided games can have observational nature, in which evaluators or experts can quantify the different actions for a specific situation (fluency), or also how many different types of solutions were generated for these situations (flexibility), or how appropriate and rare these solutions were (originality). In this sense, both game observation and game test situations (GTS) stand out as notation procedures. Memmert and Roth (2003) proposed the scale to evaluate actions within GTS, in which aspects related to flexibility and originality are verified based on a classification from 1 to 10, for which there is a description of requirements of actions for each of the scalar levels. Another possibility of instrument and variables to measure creative actions is the Creativity Behavior Assessment in Team Sports - CBATS, a tool developed to measure the individual creativity in ball possession during the game performance, identifying whether an action is standardized or non-standardized, and allowing the analysis of attempts, fluency, and versatility variables, expanding creativity measures (Santos et al., 2017, 2018). There is also the perception that the production of actions from more than one category (a large repertoire of actions) can be considered as variability, consisting of a criterion for evaluating creativity (Caso and van der Kamp, 2020).

Considering the studies included in the present review, the notational analysis presented by Santos et al. (2020) measured the frequency of passes, dribbles and shots, which were divided into success and failure. In this way, unsuccessful actions could be framed as failures or attempts, while successful actions consists the distinction between fluency and versatility. For this last classification, the observational matrix included the existence of criteria that determine whether an action is standardized or non-standardized, respectively.

Furthermore, Caso and Van der Kamp (2020) considered an observational matrix with a range of actions with and without the ball, divided among different arrangement. Variability could be verified based on the number of different categories of actions explored. Regarding the measure of originality, actions performed by approximately 5% of players or less received this rating. When the behavior was correct and appropriate, it was considered creative solution.

Studies based on the ecological premise argue that the game consists of dynamic social structures and that coordination emerges from interaction between system components. These investigations tend to be developed with other measures and, consequently, other instruments for assessing creativity. Variability and unpredictability are constructs that gain prominence and are assessed through entropy measures, whose calculation allows understanding the disorder or uncertainty measure in the system. Low entropy values reflect decrease in unpredictability, while high values indicate increase in the minimum information needed to describe the system and, consequently, greater variability (Silva et al., 2016). Another variable related to creativity is exploratory behavior, which considers the breadth of the variety of exploratory responses generated in a given system, as well as its exploration rate, which can be obtained through information extracted from global positional tracking systems, such as team amplitude and depth, centroid angle, centroid velocity, centroid distance from the goal, among others (Canton et al., 2020), or also focused on quantifying the technical-tactical actions of attackers with and without the ball and of defenders, performed within small-sided games to obtain exploration values from an observational matrix (Torrents et al., 2016).

Regarding measures of collective exploratory behavior during games, Torrents et al. (2016), Canton et al. (2019), and Canton et al. (2020) analyzed the average dynamic overlap, whose measurement allows capturing the average similarity between configurations or game patterns with each increase in the determined time distance. In this way, it becomes possible to detect measurements of the rate and breadth of exploration over different time scales.

### 4.2. Number of players in small-sided soccer games and the impact on tactical behavior

Variations of small-sided games in soccer can induce different tactical, technical, physical, and physiological responses in players (Praça et al., 2022). Regarding the tactical dimension, literature review and meta-analysis studies on convergent behavior in small-sided soccer games have already been developed (Sarmento et al., 2018; Clemente et al., 2022). In this line, studies have concluded that the manipulation of this constraint leads to the emergence of new patterns of tactical behavior and interactions among players (Ometto et al., 2018). This is observed in a primary study comparing 3 v 3 and 6 v 6 small-sided games, where the smaller configuration generates an environment in which players perform more aggressive actions, seeking more movements toward the opposing goal and one-on-one duels (Silva et al., 2014), which can be explained by the decrease in the number of collective possibilities to solve problems and the need to enhance individual skills as a resource for solving different game scenarios.

Regarding creative actions (divergent tactical behavior), the results in literature so far indicate the possibility of smaller formats of small-sided games being more favorable. Ric et al. (2016) highlighted how manipulating the numerical ratio between teams in small-sided games produce changes decision-making and tactical aspects, as the increase in the number of opponents within the game results in decrease in exploratory breadth, unpredictability, and flexibility. The analysis conducted by Torrents et al. (2016) focused on observable motor behavior, finding that players seem to show more exploratory behavior when playing with numerical disadvantage. Disadvantage forces players to vary the game, while numerical advantage seems to produce less exploration and variety. However, it is evident that playing in an unbalanced way can change the exploratory behavior of players (Canton et al., 2019). The study by Caso and van der Kamp (2020) advances in this direction and, although studies on creativity are still scarce, it suggests that the dynamics in smaller small-sided games lead players to produce more tactical actions of large number of different categories, resulting in more original and creative actions. What is also subject to discussion is that, in the aforementioned study, the 5 v 5 format stood out over larger formats that also used larger field dimensions (7 v 7 and 11 v 11); however, there was no significant difference in the number and variability of actions for the 6 v 6 format in which the field size was the same, leading us to believe that this is also a variable to be investigated.

### 4.3. Pitch size and manipulation of targets

The dimensions of the field in small-sided games represent an important object of study for tactical actions, as they provide the delimitations of the playing space and influence the space and time for solving a given problem. Ometto et al. (2018) analyzed how manipulating the field size influences tactical behaviors related to positional relationships and concluded that reducing the playing space favors the closeness between players on the same team, increases the amount of dribbling, and makes decision-making more difficult. However, a study by Clemente et al. (2022), while corroborating the idea of player proximity in smaller fields, synthesizes results regarding technical aspects and presents ambiguous data, with some studies showing better dribbling values for smaller fields while others attribute more expressive values to larger small-sided games, attributing the differences to the procedures of primary studies using different protocols.

What seems to be a consensus is that, in the context of soccer training, finding the most appropriate formatting can promote in players an adaptation that favors affordances (opportunities to act) in various situations within the task (Rico-González et al., 2022). In this sense, and more focused on providing an environment that enhances creative actions, the present study sought a calculation of the relative area per player (calculated as the field area divided by the number of players involved in the game), as variations in this relative space can change athletes’ responses (Clemente et al., 2022). The previous study by Vilar et al. (2014) aimed to understand whether altering the dimensions of the field in 5 v 5 games has the potential to shape opportunities for ball possession, passing to teammates, and shooting at the goal. Small (28 × 14 m; 39.20 m2/player), medium (40 × 20 m; 80.00 m2/player), and large (52 × 26 m; 135.20 m2/player) field configurations were used in matches, finding that a decrease in playing space provides better opportunities for ball possession, without influencing passing and shooting to teammates. Supporting the information that larger game configurations result in environment with lower number of individual player actions, other studies have also found differences in passing and shooting (Casamichana and Castellano, 2010; Owen et al., 2014). Considering that creative actions in studies largely evaluate actions related to the offensive phase, the literature seems to indicate the importance of smaller structures.

Corroborating the information previously presented, the additional analysis of the relative area per player in studies included in this review demonstrated that smaller structures, such as 5 v 5 (64.80 m2/player) and 6 v 6 (54.00 m2/player), provided an environment with higher absolute values of original and creative actions when compared to 7 v 7 (69.43 m2/player) and 10 v 10 (336.00 m2/player) games (Caso and van der Kamp, 2020). Based on results of the aforementioned study, reducing the number of players seems to favor creativity. Regarding the relative area per player, the comparison between 336.00 m2/player, 69.43 m2/player, and 54.00 m2/player seemed to indicate a trend toward increasing creativity as the field measures per player decreased. However, the smallest relative area (54.00 m2/player) for the 6 v 6 match did not present more creative and original actions than the 5 v 5 match with larger relative area (64.80 m2/player), making this assumption inconclusive. Based on these findings, it is important to consider the reduction of the relative area per player together with the numerical composition of reduced matches. Additionally, the study by Santos et al. (2020) also shows the possibility of obtaining better creativity measures by manipulating ball type, relying more on smaller space (4 v 4 with 218.75 m2/player) than on larger one (6 v 6 with 229.33 m2/player), also considering in this study that the increase in the number of players may be a significant factor.

Another relevant point is the manipulation of goals. As already mentioned, the constraints related to the number of players and field dimensions in small-sided games have potential to alter variables related to the positioning of these players, such as centroid, dispersion, and distance between players. In a 5 v 5 context, Canton et al. (2020) identified that by positioning the goals at different ends of the field, the measures obtained through tracking the movements within the playing field become distinct, relating the findings to the possibility of using it as a tool for actions different from usual, increasing the potential for originality present in small-sided game environments.

### 4.4. Variability and differential learning environments in deliberate practice

Based on the premise that variability is related to the emergence of creative movements since innovative and appropriate actions can arise from variation in the manipulation of the environment and task, inviting individuals to explore different ways of adapting to constraints, the study by Caso and van der Kamp (2020) clarifies that small-sided games tend to favor players exploring more actions.

Similarly, Santos et al. (2020) added another variability strategy to two formats of small-sided games. Based on other studies that have shown the importance of training with balls of different sizes and weights to improve motor skills, their study did not find improvement in variables related to creativity when playing small-sided games with balls other than those specific to the sport of soccer. Although the authors themselves recognize the importance of previous studies that, by analyzing longitudinally and through a training program with less habitual dynamics, found improvements in tactical actions, decrease in creativity indicators when using different ball types should not discourage this approach. The approximation of characteristics of a non-linear differential learning pedagogy and, consequently, the shift from a traditional view should consider the importance of error in skill acquisition, not focusing on correction and repetition as sources of learning, and providing environment with infinite technical variations of movement in order to make the individual able to deal with changes in games (Schollhorn et al., 2012).

A long-term deliberate practice based on games with high variability is beneficial. The study by Coutinho et al. (2018) involved young players from under-15 and under-17 categories and found improvements in physical, technical, tactical, and creative aspects for groups that participated in a physical literacy training program (control) as well as for differential learning (experimental) groups, with the second group using different balls throughout the program among other manipulations to increase variability. Furthermore, the study found better creativity scores for the experimental group in the under-15 category, corroborating stages described in the creativity development model proposed by Santos et al. (2016), whose age group (13–15 years) corresponds to the creator stage and precedes the emphasis on actions focused on specialization, and emphasizes the importance of diversification in the process. The study by Santos et al. (2018) with under-13 and under-15 categories confirms a tendency toward improvement in fluency, originality, flexibility measures, and elaboration for groups exposed to a training program that provides environment with greater need for adaptation and variations by practitioners.

### 4.5. Practical applications and study limitations

The current paper systematically brings together preliminary information and evidence that allow better knowledge of the state of the art, based on research and tests that evaluate the creativity in small soccer games, to provide interested teachers, coaches, and researchers in the thematic subsides for the choice of more accurate game configurations within the training processes.

This study has some limitations. First, in relation to the lower number of studies found that do not demonstrate an existing reproducibility of evidence synthesized so that they can be indicated as a practical solution to the central problem of this research. The second point is that the creativity measures found in different studies were also varied, which does not allow grouping and comparing them. Finally, regarding the characteristics of studies, although two were developed with young players, one had professional players as participants, making studies heterogeneous in this sense.

For future research, it is recommended to consider the importance of the game format in creativity, also highlighting the relationship between the dimensions of the field and the number of players occupying the space. Additionally, other task constraints can be explored and combined, such as manipulating the type of ball, modifying the rules of the game, varying the number and positioning of targets, limiting the number of touches on the ball, the presence of jokers, among other structural and functional modifications to the game.

## 5. Conclusion

The number of players is an important variable to consider when setting up small soccer games aimed at providing favorable environments for the emergence of creative movements. The individual, the main agent of tactical decisions within the game, must be offered situations that make it easier to increase the number of actions performed, the variability of actions and, consequently, the number of original and creative movements. In this sense, small-sided games with fewer components seems to be more favorable than large spaces and more players, with decreases in creativity measures being found as the game configuration increases. The relative area per player still needs to be further investigated, but it seems to be an important component to consider when thinking about the tactical scenarios of small-sided games and their influence on the emergence of creative actions. In addition, it is possible to perceive that other manipulations can be inserted in the context of reduced games, with the intention of influencing tactical and technical behaviors, establishing regulations that in the long term can be important to diversify and increase the creative repertoire of players.

## Data availability statement

The original contributions presented in the study are included in the article/supplementary material, further inquiries can be directed to the corresponding author.

## Author contributions

LU: Conceptualization, Data curation, Formal analysis, Investigation, Methodology, Writing – original draft. MM: Conceptualization, Data curation, Formal analysis, Writing – original draft, Writing – review & editing. GP: Data curation, Formal analysis, Investigation, Project administration, Writing – original draft, Writing – review & editing. GM: Data curation, Investigation, Writing – original draft, Writing – review & editing. JS: Conceptualization, Formal analysis, Investigation, Methodology, Supervision, Writing – review & editing. PB: Conceptualization, Data curation, Investigation, Methodology, Supervision, Writing – original draft.
